# Optimizing artificial insemination in goats: semen deposition site and vaginal mucus characteristics as predictive biomarkers

**DOI:** 10.3389/fvets.2025.1667834

**Published:** 2025-10-24

**Authors:** Ting-Chieh Kang, Kai-Fei Tseng, Hsin-Hung Lin, Tsai-Tzu Chen, I-Ling Lai, Perng-Chih Shen

**Affiliations:** 1 Southern Branch, Taiwan Livestock Research Institute, Ministry of Agriculture, Pingtung, Taiwan; 2 Graduate Institute of Bioresources, National Pingtung University of Science and Technology, Pingtung, Taiwan; 3 Department of Animal Science, National Pingtung University of Science and Technology, Neipu, Pingtung, Taiwan

**Keywords:** artificial insemination, biomarkers, fertility, alpine goats, semen deposition site, vaginal mucus

## Abstract

Artificial insemination (AI) is a critical tool for genetic improvement and fertility management in goats. This study examined the effects of semen deposition site (uterine body, cervix, vagina) and vaginal mucus type (cloudy, turbid, clear) on pregnancy rate, kidding rate, and average litter size in 300 CIDR-synchronized Alpine does in southern Taiwan. Semen deposited in the uterine body combined with cloudy mucus yielded the highest pregnancy rate (55.9%), while vaginal deposition with clear mucus resulted in the lowest (30.7%). Two-way ANOVA showed significant main effects and interactions for pregnancy rate and average litter size (*p* < 0.05), but no significant effect on kidding rate. Pregnant does exhibited lower vaginal mucus electrical conductivity, higher pH, and elevated temperature compared to non-pregnant does, suggesting these parameters as potential biomarkers for estrus detection. Findings highlight the importance of precise semen placement and optimal mucus condition for improving AI protocols in goats.

## 1 Introduction

Artificial insemination (AI) is one of the most significant reproductive biotechnologies in modern goat production. It plays a critical role in genetic improvement, enhancing productivity, and enabling more effective management of breeding programs (1–3). Through AI, farmers can access superior genetic resources from elite bucks, resulting in offspring with higher milk yield, faster growth, and greater disease resistance. Moreover, AI reduces the risk of disease transmission and allows strategic planning of breeding seasons, thereby supporting the sustainable development of livestock systems (4–6).

Despite its advantages, the success of AI is influenced by multiple biological and environmental factors, among which semen deposition site and vaginal mucus characteristics are especially important (7). Previous studies have consistently shown that deeper semen deposition within the female reproductive tract is associated with higher pregnancy rates. Laparoscopic intrauterine insemination (LAI), which directly deposits semen into the uterine horns, typically achieves the highest success rates (8–10), followed by transcervical insemination, while vaginal insemination yields the lowest (11–13). This occurs because closer semen deposition to the fertilization site reduces the distance sperm must travel and the physiological barriers they encounter, consistent with current models of sperm-tract interactions (14–16).

Beyond deposition site, vaginal and cervical mucus characteristics including appearance, consistency, pH, and electrical conductivity vary throughout the estrous cycle and directly influence sperm survival and transport (17, 18). Cervical mucus is generally clear and fluid during estrus, facilitating sperm passage, but becomes increasingly viscous after estrus, which restricts sperm motility (18–20). Vaginal electrical resistance (VER) typically declines around estrus, making it a useful adjunct for AI timing. Recent work has provided reference ranges for goats and supports the use of electrical measures as objective biomarkers (21, 22). In addition, external factors such as ambient temperature and meteorological conditions have been shown further to modulate fertility following AI (23–25).

Although these parameters are well documented as indicators for estrus detection and ovulation timing, their potential as predictive biomarkers at the actual moment of insemination remains underexplored. Most prior research has focused on determining whether a doe is in estrus and estimating ovulation rather than systematically documenting mucus appearance and physicochemical properties such as conductivity, pH, and temperature synchronously with AI procedures. As a result, there is a lack of systematic field data directly linking these real-time measurements to reproductive outcomes, including pregnancy rate, kidding rate, and litter size. This gap limits the development of practical, science-based tools that could enhance AI efficiency under commercial farm conditions.

This study aimed to evaluate how semen deposition site and vaginal mucus characteristics influence AI outcomes in Alpine goats and determine whether mucus physicochemical properties (pH, conductivity, temperature) can serve as practical biomarkers for optimal insemination timing. By integrating anatomical precision with real-time physiological assessment, we sought to establish evidence-based guidelines for improving reproductive management in commercial goat production systems.

## 2 Materials and methods

### 2.1 Animals

#### 2.1.1 Does

Field data from 300 Alpine does inseminated was collected in this study. The animals were sourced from three private farms in southern Taiwan, namely Kaixiang dairy goat farm in Zhongpu, Chiayi, Songjun livestock farm, and Jiata farm. The data collection period focused on the breeding season from October to November of 2024. All does were between 2 and 3 years of age and had given birth 1 to 2 times. The average daily milk yield ranged from 2.3 to 2.7 kg.

The 300 does were distributed across nine treatment combinations (3 deposition sites × 3 mucus types) as follows: uterine body deposition (*n* = 84: cloudy *n* = 28, turbid *n* = 37, clear *n* = 19), cervical deposition (*n* = 155: cloudy *n* = 42, turbid *n* = 67, clear *n* = 46), and vaginal deposition (*n* = 61: cloudy *n* = 15, turbid *n* = 23, clear *n* = 23). The uneven distribution reflects the natural occurrence of different mucus types and the technical challenges associated with achieving deeper semen deposition in field conditions.

Importantly, no grouping of animals was conducted at the time of data collection. Vaginal mucus observations, including electrical conductivity, pH, and vaginal temperature, were measured and recorded on-site at the time of insemination. Subsequently, the dataset was classified retrospectively according to pregnancy rate, kidding rate, and average litter size.

#### 2.1.2 Bucks and semen source of frozen semen and artificial insemination

The frozen semen for artificial insemination (AI) was purchased from the Southern Branch of Livestock Research Institute, Council of Agriculture Taiwan and one buck was the origin of semen. Each straw contained 0.5 mL of semen with a concentration of 150 × 10⁶ sperm/0.5 mL. Only seminal samples with a post-thaw sperm motility >50% along with a progressive motility >40% were selected. The buck was 4 years 10 months old and had sired 2,200 doses of frozen semen.

#### 2.1.3 Preparation of semen and composition of the extender

Semen straws were thawed in a 37 °C water bath for 30 s before use. Semen quality at post-thaw was analyzed using a computer-assisted sperm analyzer (Sperm Class Analyzer® CASA System, MICROPTIC, Barcelona, Spain). Semen with ≥50% total motility and ≥50% progressive motility were only included. The semen extender was formulated per 100 mL as follows: 2.42 g Tris (hydroxymethyl aminomethane; T1503), 1.48 g citric acid (C0759), 1.00 g glucose (G8270), 1 mL penicillin–streptomycin solution (P4333), 6% low-density lipoprotein (LDL) on a dry matter basis. The concentration of glycerol (G5516) was set to 7% (v/v). All chemicals were obtained from Sigma-Aldrich.

### 2.2 Estrus synchronization application to does

All does were synchronized using a Controlled Internal Drug Release device (CIDR®; EAZI–breed, Rydalmere, Australia) in combination with pregnant mare serum gonadotropin (PMSG, Prospec-Tany, Israel) and a prostaglandin F2α analog (cloprostenol, Estrumate®, Merck Animal Health, United States). CIDR® was administered intravaginally on Day 0. An intramuscular injection of PGF2α (5.3 mg/0.5 mL) and PMSG (500 IU) was performed on Day 9. CIDR® was withdrawn at Day 11 and AI was performed 40 h after CIDR withdrawal.

### 2.3 Measurement and assessment criteria of all parameters

#### 2.3.1 Pregnancy rate (%)

Pregnancy was determined 45 days after artificial insemination by transrectal ultrasonography with an ultrasound scanner (Aloka SSD-500, Japan) and a transrectal linear probe (3.5 MHz). Pregnancy was diagnosed by recognition of uterine horn shapes and images of fetuses.

#### 2.3.2 Kidding rate (%)

The proportion of does conceived around 150 ± 7 days post artificial insemination.

#### 2.3.3 Average litter size-kid

Number of kids born/Number of does that kidded.

### 2.4 Artificial insemination process

#### 2.4.1 Semen deposition site

**Figure 1:**
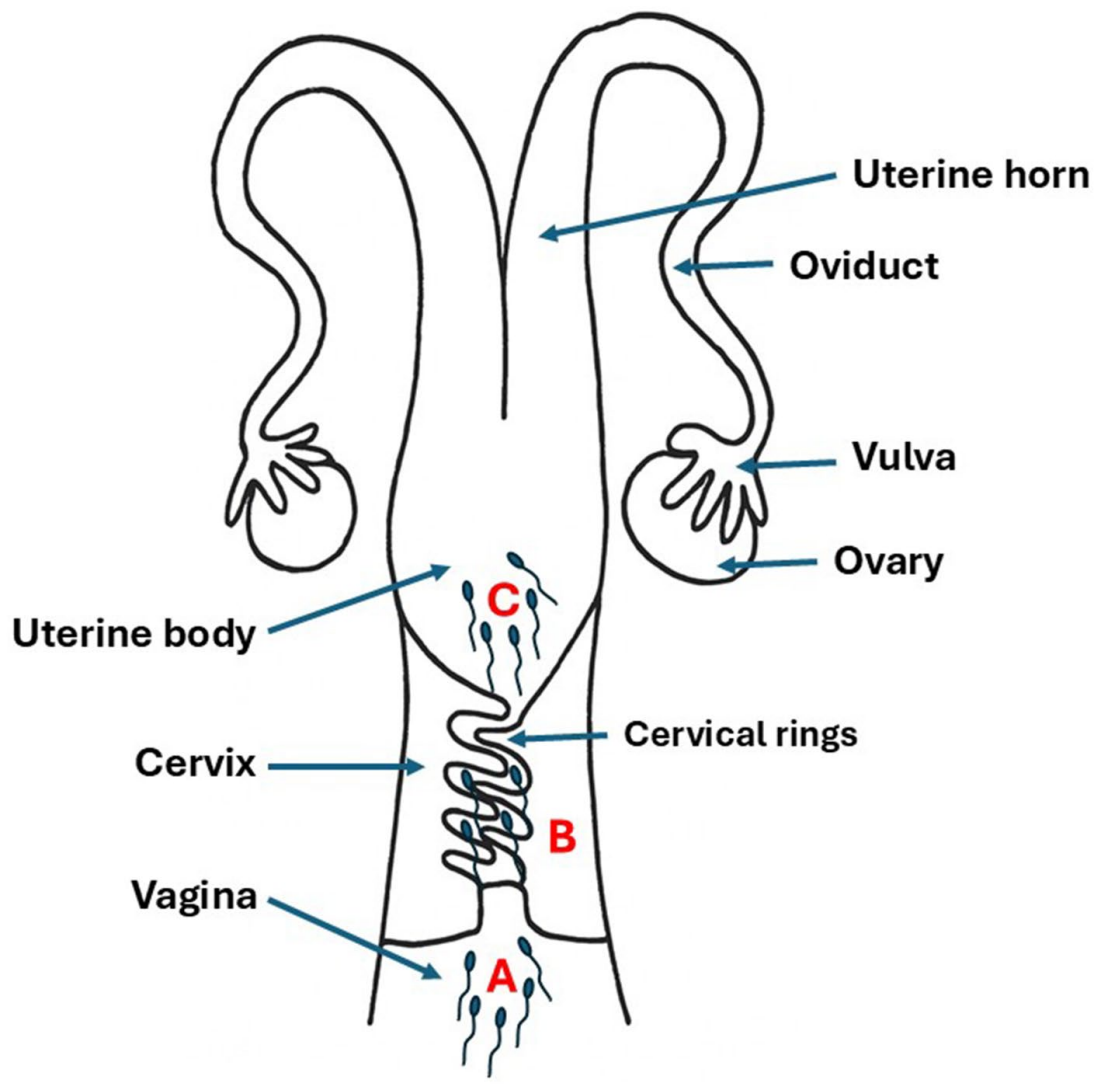
Schematic representation of semen deposition sites during artificial insemination in goats. **(A)** Vaginal deposition: semen placed in vaginal cavity without cervical penetration. **(B)** Cervical deposition: semen deposited 2 cm into cervix through cervical rings. **(C)** Uterine body deposition: semen placed directly into uterine body through complete cervical passage. Arrows indicate semen placement locations.

The inseminator categorized semen deposition sites into 3 distinct types according to the depth of sheathization and tactile sensation (Figure 1):

- Vaginal: An insemination sheath was inserted through the external cervical, but the tip was not inserted into the cervix.
- Cervical: The artificial insemination sheath was inserted approximately 2 cm into the cervix, the tip of the sheath partially through the screw circle the cervix, but not into the uterine body, when injection, no reflux of the semen back to the vagina.
- Body of the uterus: The sheath entered the cervix, and ejaculate was placed directly into the body of the uterus.

#### 2.4.2 Vaginal mucus type

**Figure 2:**
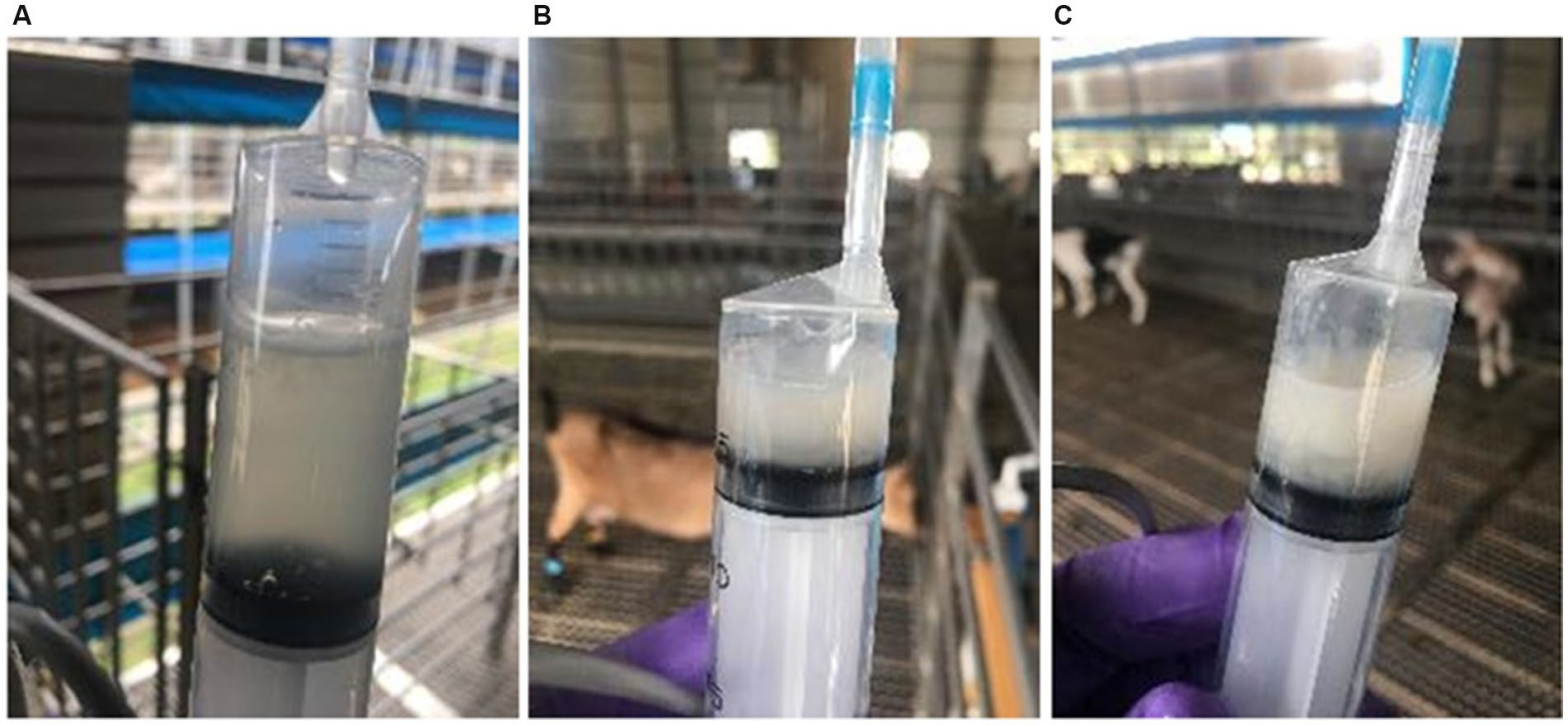
Vaginal mucus types of artificial insemination. **(A)** Clear, **(B)** Turbid, **(C)** Cloudy.

Prior to insemination, vaginal mucus was evaluated visually by the technician, with the aid of a speculum and sorted into 3 categories (Figure 2):

- Clear: Transparent with good fluidity, no noticeable odor or suspended particles.
- Turbid: Opaque with flocculent substances, moderate fluidity.
- Cloudy: Milky and viscous, poor fluidity often adheres to the vaginal wall.

The combination of the two factors (site of semen deposition × type of mucus) resulted in a 3 × 3 factorial arrangements consisting of nine observations.

### 2.5 Measurement of vaginal mucus conductivity, pH, and vaginal temperature and personnel

#### 2.5.1 Personnel

All artificial insemination procedures were performed by the same operator to minimize subjective variability in assessing vaginal mucus characteristics and semen deposition sites.

#### 2.5.2 Measurement of vaginal mucus conductivity, pH, and temperature

During artificial insemination, the physical properties of vaginal mucus were measured using a 3-in-1 detector (TES–1381) manufactured by TES Electrical Electronic Corp. The pH measurement had a resolution of 0.001 and an accuracy of ± 0.01. Conductivity resolution ranged from 0.001 μS/cm to 0.01 mS/cm, with accuracy between ± 2% and ± 5% of full scale (FS). Temperature resolution was 0.1 °C or 0.1 °F, with an accuracy of ± 0.5 °C for Celsius measurements and ± 0.9 °F for Fahrenheit.

### 2.6 Sperm motility assessment

Prior to use, all frozen semen samples were thawed and assessed for motion and progressive movement through Object Computer-Assisted Sperm Analysis (CASA). Only samples with post-thaw motility >50% and progressive motility >40% were considered.

### 2.7 Artificial insemination procedure

The frozen semen straws were obtained from storage in liquid nitrogen, and were suspended in the water bath at 37 °C and thawed for 30 s. Upon thawing, the straw was dried with tissue paper, the seal severed, and the semen transferred into the insemination gun. A lubricated speculum (K-Y jelly, Johnson and Johnson Medical, Ltd., Gargrave, Skipton, BD23 3RX, United Kingdom), was carefully placed in the vagina, its narrow end first and then it was rotated 90° to view the cervical, prior to insemination. The insemination gun was advanced through the cervix; resistance to the passage of the gun was noted, and insertion was halted, and insemination was conducted to prevent tissue damage or immunological reactions along with likely reduction in conception percentage.

### 2.8 Statistical analysis

All statistical analyses were performed by SAS® Enterprise Guide version 7.4 (SAS Institute Inc., Cary, NC, United States). Values are expressed as the mean ± SE. Pregnancy rates were tested among semen deposition sites and mucus types using one-way ANOVA followed by Tukey’s Honestly Significant Difference (HSD) *post-hoc* analysis. Means with different lowercase letters (a, b, c) within the same column are significantly different (*p* < 0.05).

Multiple linear regression and two-way analysis of variance (Two-way ANOVA) were conducted to evaluate the effects of cervical mucus condition and semen deposition location on pregnancy rate, kidding rate, and average litter size.

In the multiple linear regression analysis, cervical mucus condition and semen deposition location were coded as ordinal variables (mucus: 1 = clear, 2 = turbid, 3 = cloudy; location: 1 = vagina, 2 = cervix, 3 = uterine body) to explore whether a monotonic relationship exists across levels. This approach does not assume strict linearity but provides an initial assessment of potential trend-like associations (e.g., increasing mucus opacity or deeper semen deposition site may correlate with altered reproductive outcomes).

Although mucus condition and semen deposition site were coded numerically (1–3) for regression analysis, both variables remained categorical in nature. This ordinal coding was employed to test monotonic trends across treatment levels rather than to assume strict linearity. The consistency between ordinal regression and categorical ANOVA results validates the robustness of our statistical approach and confirms that observed associations reflect genuine biological relationships rather than methodological artifacts.

To address potential concerns regarding the assumption of linearity, the same factors were also analyzed as categorical variables in the two-way ANOVA model. Tukey’s *post-hoc* test was applied for multiple comparisons where appropriate. The consistency of results across both analytical approaches was used to validate the robustness of the findings. Statistical significance was set at *p* < 0.05.

Sample size calculations were based on detecting a 20% difference in pregnancy rates between treatment groups with 80% power and *α* = 0.05. *Post-hoc* power analysis confirmed adequate statistical power (>85%) for detecting the observed effect sizes in pregnancy rate and litter size outcomes.

## 3 Results

This study evaluated the influence of semen deposition sites (uterine body, cervical, and vaginal) and vaginal mucus characteristics (cloudy, turbid, and clear) on reproductive performance in goat, including pregnancy rate, kidding rate, and average litter size following artificial insemination.

### 3.1 Effects of semen deposition site and mucus characteristics on reproductive efficiency

The results showed in Table 1. Both semen deposition site and mucus type significant affected pregnancy rate and litter size (*p* < 0.05), whereas kidding rate remained stable across treatments (*p* > 0.05). Pregnancy outcomes were consistently higher when semen was deposited deeper within the reproductive tract (uterine body or cervix) and when mucus was more viscous (cloudy or turbid).

**Table 1:** Artificial insemination semen deposition location, mucus status and pregnancy rate in goats.

Position	Mucus condition	n	Pregnancy rate %	Kidding rate %	Average litter size
Uterine body	Cloudy	28	55.85 ± 0.77ᵃ	89.80 ± 0.26ᵃ	1.78 ± 0.01ᵃ
Uterine body	Turbid	37	53.20 ± 0.67ᵇ	89.65 ± 0.32ᵃ	1.72 ± 0.01ᵃᵇ
Uterine body	Clear	19	44.12 ± 0.45ᵈ	89.70 ± 0.29ᵃ	1.60 ± 0.03ᵇᶜᵈ
Cervical	Cloudy	42	51.38 ± 0.50ᵇ	89.75 ± 0.30ᵃ	1.68 ± 0.03ᵃᵇᶜ
Cervical	Turbid	67	48.55 ± 0.52ᶜ	90.05 ± 0.27ᵃ	1.81 ± 0.01ᵃ
Cervical	Clear	46	39.23 ± 0.38ᵉ	89.50 ± 0.35ᵃ	1.60 ± 0.03ᵇˢᵈ
Vaginal	Cloudy	15	45.77 ± 0.56ᵈ	89.00 ± 0.34ᵃ	1.58 ± 0.03ᶜᵈ
Vaginal	Turbid	23	41.48 ± 0.39ᵉ	89.65 ± 0.29ᵃ	1.51 ± 0.05ᵈ
Vaginal	Clear	23	30.68 ± 0.42ᶠ	89.30 ± 0.34ᵃ	1.31 ± 0.03ᵉ

Pregnancy rate (%) was calculated using the total number of inseminated does as the denominator. Kidding rate (%) was calculated using the number of confirmed pregnant does as the denominator. Values are expressed as means ± standard error (SE). n represents the number of does. ^ᵃ, ᵇ, ᶜ, ᵈ, ᵉ, ᶠ^ Different letters within the same column indicate statistically significant differences between groups (*p* < 0.05).

The uterine body–cloudy combination achieved the highest pregnancy rate (55.9, 95% CI, 54.4–57.4), significantly outperforming most other combinations. By contrast, the lowest rate occurred in the vaginal–clear mucus group (30.68, 95% CI, 29.84–31.52).

### 3.2 Correlation between vaginal mucus characteristics, semen deposition site, and reproductive performance

Regression and two-way ANOVA analyses (Table 2) demonstrated that pregnancy rate was the most responsive outcome to both experimental factors, followed by litter size, while kidding rate showed little variation.

**Table 2:** Effects of cervical mucus and semen deposition site on reproductive outcomes in goats.

Outcome	Analysis	Factor	Estimate / *F*-value	*p*-value
Pregnancy rate	Regression	Mucus score	6.50	<0.0001
		Deposition site	5.88	<0.0001
	Two-way ANOVA	Mucus	483.06	<0.0001
		Deposition site	370.00	<0.0001
		Interaction	2.98	0.019
Kidding rate	Regression	Mucus score	0.04	0.834
		Deposition site	0.41	0.683
	Two-way ANOVA	Mucus	1.24	0.277
		Deposition site	2.13	0.132
		Interaction	2.22	0.068
Average litter size	Regression	Mucus score	0.18	<0.0001
		Deposition site	0.25	<0.0001
	Two-way ANOVA	Mucus	27.24	<0.0001
		Deposition site	33.42	<0.0001
		Interaction	4.60	0.003

#### 3.2.1 Pregnancy rate

Mucus score (estimate = 6.50, *p* < 0.0001) and deposition site (estimate = 5.88, *p* < 0.0001) were both strongly associated with improved outcomes, with a significant interaction effect (*F* = 2.98, *p* = 0.019).

#### 3.2.2 Kidding rate

Neither mucus quality nor deposition site had significant effects (*p* > 0.05), although the interaction approached significance (*p* = 0.068).

#### 3.2.3 Average litter size

Both factors contributed positively to offspring number (mucus estimate = 0.18; site estimate = 0.25; both *p* < 0.0001), with a significant interaction effect (*F* = 4.60, *p* = 0.003).

These results emphasize that optimizing mucus quality (favoring turbid or cloudy) and semen deposition site (uterine body or cervix) produces synergistic improvements in conception probability and litter size, while exerting limited influence on kidding success.

### 3.3 Vaginal mucus electrical conductivity, pH, and temperature between pregnant and non-pregnant goats

Physiological properties of vaginal mucus differed significantly between pregnant and non-pregnant does (Table 3). Pregnant goats consistently exhibited lower electrical conductivity, higher pH, and slightly elevated vaginal temperatures compared with non-pregnant counterparts.

**Table 3:** Vaginal mucus electrical resistance, pH, and vaginal temperature in pregnant and non-pregnant goats.

Category	Mucus condition	n	Electrical conductivity	pH	Temperature, °C
Pregnant	Cloudy	46	286.96 ± 2.99ᵈ	7.55 ± 0.03ᵃ	39.02 ± 0.03ᵃ
	Turbid	63	314.94 ± 0.23ᶜ	7.29 ± 0.02ᵇ	38.94 ± 0.02ᵃ
	Clear	32	394.53 ± 10.76ᵇ	6.57 ± 0.05ᶜ	38.38 ± 0.03ᵇ
Non-pregnant	Cloudy	39	322.05 ± 1.00ᶜ	6.40 ± 0.04ᶜ	38.95 ± 0.03ᵃ
	Turbid	64	325.16 ± 1.04ᶜ	6.50 ± 0.04ᶜ	38.95 ± 0.02ᵃ
	Clear	56	427.67 ± 10.16ᵃ	6.53 ± 0.05ᵃ	38.30 ± 0.03ᵇ

Values are expressed as means ± standard error (SE). n represents the number of does. EC, Electrical Conductivity (μS/cm). ^ᵃ, ᵇ, ᶜ, ᵈ^ Different letters within the same column indicate statistically significant differences between groups (*p* < 0.05).

#### 3.3.1 Electrical conductivity

Pregnant does had the lowest EC in the cloudy mucus group (~287 μS/cm), whereas non-pregnant does exhibited markedly higher EC in the clear mucus group (~428 μS/cm).

#### 3.3.2 pH

Vaginal mucus in pregnant does was more alkaline, reaching pH 7.55 in cloudy mucus, compared with 6.40–6.53 in non-pregnant does.

#### 3.3.3 Temperature

Pregnant does maintained higher vaginal temperatures (~39.0 °C) under turbid and cloudy conditions, while non-pregnant does exhibited slightly lower temperatures (~38.3–38.9 °C).

Together, these physiological markers lower EC, higher pH, and elevated temperature in viscous mucus may serve as reliable indicators of successful conception in goats.

## 4 Discussion

### 4.1 Effect of semen deposition site on artificial insemination success rate in goats

The present findings clearly demonstrate that the anatomical site of semen deposition has a decisive effect on AI success in goats. Pregnancy rates were consistently higher when semen was deposited in the uterine body compared with the cervix or vagina, particularly under favorable mucus conditions. For example, uterine body–cloudy and uterine body–turbid groups yielded conception rates exceeding 53%, whereas vaginal-clear inseminations achieved less than 31%. These observations corroborate earlier studies highlighting the advantage of deeper semen placement in small ruminants (26, 27).

Leethongdee et al. (28) further reported that semen retained in the vagina without pretreatment reduces fertility, while intrauterine insemination enhances outcomes (29) similarly showed that laparoscopic AI with frozen semen can achieve 60–80% pregnancy rates, compared with less than 40% using cervical or vaginal methods. Anatomically, the goat cervix contains four to five annular folds that impede catheter passage and present immune challenges to sperm survival (30, 31, 32). Consistent with large-scale field studies in Spanish goat breeds, accurate and deeper deposition remains central to maximizing fertility (23, 24, 33).

### 4.2 Influence of vaginal and cervical mucus characteristics on reproductive success

Mucus properties represent a critical physiological biomarker of estrus and directly influence insemination success. In hormonally synchronized does, clear mucus is typically observed under conditions of high estrogen dominance. This type of mucus is characterized by high water and electrolyte content and low viscosity, which facilitates sperm penetration but offers limited protection against the acidic vaginal environment (26, 27).

As ovulation approaches and the luteinizing hormone (LH) surges, mucus composition shifts toward higher concentrations of glycoproteins, particularly mucins, as well as cellular debris. These changes result in turbid or cloudy appearances with increased viscosity and stronger adhesion to the vaginal wall (27, 28). While this increased viscosity may restrict sperm motility, it also provides a more protective environment that enhances sperm survival (29).

Our findings confirmed this physiological pattern: conception rates were highest when mucus was turbid, intermediate when it was cloudy, and lowest when it was clear. These results align with earlier reports indicating that insemination timed at the transition to turbid mucus closely predicts ovulation (28, 29). Conversely, although clear mucus is strongly associated with estrogen, it may obstruct the cervix and complicate insemination procedures (30).

From a practical standpoint, artificial insemination protocols that consider mucus quality can improve reproductive outcomes. Monitoring the transition from clear to turbid/cloudy mucus provides a valuable visual biomarker of imminent ovulation for field inseminators. Moreover, adjunct strategies such as the use of hyaluronic acid to facilitate catheter passage may further enhance insemination success and fertility outcomes (28, 29).

### 4.3 Interaction effects of semen deposition site and mucus characteristics

Our factorial analysis revealed that semen deposition site and mucus characteristics act synergistically to determine fertility outcomes. Both pregnancy rate and litter size were significantly influenced by the combination of these two variables, while kidding rate remained unaffected.

Methodological considerations. Beyond the biological interpretation, several methodological aspects deserve clarification. In the regression models, cervical mucus condition and semen deposition site were treated as ordinal variables coded from 1 to 3. While this might raise concerns about the assumption of linearity, our intention was to test monotonic trends across levels rather than to imply strict equal-interval scaling. For example, progressive increases in mucus opacity or deeper deposition sites may reasonably be expected to produce directional changes in reproductive outcomes. To address potential concerns, we also analyzed these variables as categorical factors using two-way ANOVA. The consistency between the ordinal and categorical analyses supports the robustness of our findings and strengthens the argument that the associations observed are biologically meaningful rather than statistical artifacts.

Main effects. Cervical mucus emerged as the strongest determinant of pregnancy rate and litter size, with higher-quality mucus consistently associated with improved fertility. The effect of semen deposition site was also clear, with deeper placements leading to higher conception probabilities—an effect that parallels observations in sheep, where each additional centimeter of insemination depth enhances pregnancy likelihood (26, 27).

Interaction effects. The significant interactions revealed in our study indicate that optimal fertility requires both high-quality mucus and precise deposition. When mucus is suboptimal, accurate anatomical deposition becomes crucial, whereas favorable mucus quality can partly offset less precise semen placement.

Kidding rate. Interestingly, neither deposition site nor mucus characteristics significantly influenced kidding rate, suggesting that once conception occurs, gestation and parturition are more strongly driven by maternal health, nutrition, and environmental management factors (12, 36).

### 4.4 Vaginal electrical conductivity, pH, and temperature as complementary biomarkers

In addition to visual mucus assessment, vaginal electrical conductivity (EC), pH, and temperature were evaluated as complementary biomarkers of reproductive status. Physiological parameters varied consistently between pregnant and non-pregnant does under comparable mucus conditions. For instance, under turbid mucus, pregnant does showed an average pH of 7.55 compared with 6.40 in non-pregnant counterparts. Elevated pH provides a favorable environment for sperm survival, capacitation, and penetration, whereas acidic conditions impair motility (26, 28, 29).

Electrical measurements, including vaginal electrical resistance (VER) and EC, have been increasingly validated as reliable indicators for estrus detection in goats (30, 31). Typically, VER declines during estrus, reflecting peak estrogen activity and enhanced sperm transport conditions (29). Our findings, which demonstrated lower EC values in pregnant does—particularly under turbid and cloudy mucus—are consistent with these physiological interpretations. Although EC and VER differ technically, both capture ionic shifts in the vaginal microenvironment, and their convergence reinforces the biological relevance of these measures (29, 31).

Vaginal temperature also exhibited diagnostic potential. Pregnant does under turbid and cloudy mucus conditions maintained higher vaginal temperatures (~39.0 °C) than those under clear mucus (~38.3 °C), a pattern consistent with estrogen-induced increases in blood flow and metabolic activity (27). Furthermore, hormonal interventions such as hCG may be integrated with objective physiological markers (mucus, EC, pH, and temperature) to refine ovulation timing and improve insemination outcomes (28, 29).

In our study, electrical conductivity (EC) was measured using field-based EC meters, which directly report conductivity values (in μS/cm or mS/cm). As conductivity (*σ*) is the mathematical inverse of resistivity (*ρ*), and resistance (R) is related to resistivity via σ = 1/ρ, R = ρ·L/A, there exists a direct and well-established relationship between resistance and conductivity (21, 22).

Therefore, the use of EC in our analysis serves the same physiological and diagnostic purpose as resistance measurements—namely, characterizing the ionic and biochemical environment of vaginal mucus. The difference primarily lies in the instrumentation and unit convention, not in the biological interpretation.

From a practical perspective, the choice of EC in this study was guided by the field advantages of portable multi-parameter probes, which allow rapid and cost-effective assessment under farm conditions. Thus, combining objective biomarkers with optimized semen deposition strategies aligns with contemporary models of sperm transport, oviductal interaction, and fertilization success (30, 31).

### 4.5 Practical recommendations for field AI programs

Based on our findings, several practical recommendations can be made for optimizing AI protocols in commercial goat operations. First, prioritize semen deposition in the uterine body or deep cervix whenever technically feasible, as this consistently improves pregnancy rates by 15–25% compared to vaginal deposition. Second, time insemination coincides with cloudy or turbid mucus conditions, which can be easily assessed visually during routine AI procedures. Third, consider incorporating simple physiological measurements (pH, electrical conductivity, temperature) as supplementary tools for estrus detection and optimal timing, particularly in synchronized breeding programs. Finally, ensure adequate operator training to achieve consistent deposition depth and mucus assessment, as technical precision significantly influences reproductive outcomes. These evidence-based modifications to existing AI protocols could substantially improve fertility rates while maintaining cost-effectiveness in commercial goat production systems.

## Data Availability

The original contributions presented in the study are included in the article/supplementary material, further inquiries can be directed to the corresponding author.
