# Correction: Optimizing artificial insemination in goats: semen deposition site and vaginal mucus characteristics as predictive biomarkers

**DOI:** 10.3389/fvets.2026.1807918

**Published:** 2026-03-20

**Authors:** Ting-Chieh Kang, Kai-Fei Tseng, Hsin-Hung Lin, Tsai-Tzu Chen, I-Ling Lai, Perng-Chih Shen

**Affiliations:** 1 Southern Branch, Taiwan Livestock Research Institute, Ministry of Agriculture, Pingtung, Taiwan; 2 Graduate Institute of Bioresources, National Pingtung University of Science and Technology, Pingtung, Taiwan; 3 Department of Animal Science, National Pingtung University of Science and Technology, Neipu, Pingtung, Taiwan

**Keywords:** artificial insemination, biomarkers, fertility, alpine goats, semen deposition site, vaginal mucus

In the published article, there was an error in Sections 2.3.1 (Pregnancy rate, %) and 2.3.2 (Kidding rate, %). The insemination procedure was incorrectly described as laparoscopic artificial insemination, whereas the procedure used in this study was artificial insemination.

A correction has been made to Section 2.3.1 (Pregnancy rate, %) The sentence previously stated:

“Pregnancy was determined 45 days after laparoscopic AI by transrectal ultrasonography with an ultrasound scanner (Aloka SSD-500, Japan) and a transrectal linear probe (3.5 MHz). Pregnancy was diagnosed by recognition of uterine horn shapes and images of fetuses.”

The corrected sentence appears below:

“Pregnancy was determined 45 days after artificial insemination by transrectal ultrasonography with an ultrasound scanner (Aloka SSD-500, Japan) and a transrectal linear probe (3.5 MHz). Pregnancy was diagnosed by recognition of uterine horn shapes and images of fetuses.”

A correction has been made to Section 2.3.2 (Kidding rate, %). The sentence previously stated:

“The proportion of does conceived around 150 ± 7 days post-laparoscopic artificial insemination.”

The corrected sentence appear below:

“The proportion of does conceived around 150 ± 7 days post artificial insemination.”

In the published article, there was an error in Table 1 as published. The explanatory note did not clearly describe the calculation basis for pregnancy rate and kidding rate, which may lead to misunderstanding for readers.

The corrected Table 1 and its caption “Artificial insemination semen deposition location, mucus status and pregnancy rate in goats” and corrected footnote appear below.

**Table 1:** Artificial insemination semen deposition location, mucus status and pregnancy rate in goats.

**Position**	**Mucus condition**	***n***	**Pregnancy rate**	**Kidding rate**	**Average little size**
			**%**		
Uterine body	Cloudy	28	55.85 ± 0.77^a^	89.80 ± 0.26^a^	1.78 ± 0.01^a^
Uterine body	Turbid	37	53.20 ± 0.67^b^	89.65 ± 0.32^a^	1.72 ± 0.01^ab^
Uterine body	Clear	19	44.12 ± 0.45^d^	89.70 ± 0.29^a^	1.60 ± 0.03^bcd^
Cervical	Cloudy	42	51.38 ± 0.50^b^	89.75 ± 0.30^a^	1.68 ± 0.03^abc^
Cervical	Turbid	67	48.55 ± 0.52^c^	90.05 ± 0.27^a^	1.81 ± 0.01^a^
Cervical	Clear	46	39.23 ± 0.38^e^	89.50 ± 0.35^a^	1.60 ± 0.03^bcd^
Vaginal	Cloudy	15	45.77 ± 0.56^d^	89.00 ± 0.34^a^	1.58 ± 0.03^cd^
Vaginal	Turbid	23	41.48 ± 0.39^e^	89.65 ± 0.29^a^	1.51 ± 0.05^d^
Vaginal	Clear	23	30.68 ± 0.42^f^	89.30 ± 0.34^a^	1.31 ± 0.03^e^

Pregnancy rate (%) was calculated using the total number of inseminated does as the denominator. Kidding rate (%) was calculated using the number of confirmed pregnant does as the denominator. Values are expressed as means ± standard error (SE). *n* represents the number of does.

^a, b, c, d, e, f^Different letters within the same column indicate statistically significant differences between groups (*P* < 0.05).

The original article has been updated.

